# The effect of seasonal birth pulses on pathogen persistence in wild mammal populations

**DOI:** 10.1098/rspb.2013.2962

**Published:** 2014-07-07

**Authors:** A. J. Peel, J. R. C. Pulliam, A. D. Luis, R. K. Plowright, T. J. O'Shea, D. T. S. Hayman, J. L. N. Wood, C. T. Webb, O. Restif

**Affiliations:** 1 Disease Dynamics Unit, Department of Veterinary Medicine, University of Cambridge, Cambridge CB3 0ES, UK; 2 Institute of Zoology, Zoological Society of London, Regent's Park, London NW1 4RY, UK; 3 Environmental Futures Research Institute, Griffith University, Brisbane, 4111 Australia; 4 Fogarty International Center, National Institutes of Health, Bethesda, MD 20892, USA; 5 Department of Biology, University of Florida, Gainesville, FL 32611, USA; 6 Emerging Pathogens Institute, University of Florida, Gainesville, FL 32610, USA; 7 Department of Biology, Colorado State University, Fort Collins, CO 80523, USA; 8 Center for Infectious Disease Dynamics, The Pennsylvania State University, University Park, PA 16802, USA; 9 US Geological Survey (retired), PO Box 65, Glen Haven, CO 80532, USA

**Keywords:** critical community size, wildlife epidemiology, birth pulse, seasonality, stochastic model

## Abstract

The notion of a critical community size (CCS), or population size that is likely to result in long-term persistence of a communicable disease, has been developed based on the empirical observations of acute immunizing infections in human populations, and extended for use in wildlife populations. Seasonal birth pulses are frequently observed in wildlife and are expected to impact infection dynamics, yet their effect on pathogen persistence and CCS have not been considered. To investigate this issue theoretically, we use stochastic epidemiological models to ask how host life-history traits and infection parameters interact to determine pathogen persistence within a closed population. We fit seasonal birth pulse models to data from diverse mammalian species in order to identify realistic parameter ranges. When varying the synchrony of the birth pulse with all other parameters being constant, our model predicted that the CCS can vary by more than two orders of magnitude. Tighter birth pulses tended to drive pathogen extinction by creating large amplitude oscillations in prevalence, especially with high demographic turnover and short infectious periods. Parameters affecting the relative timing of the epidemic and birth pulse peaks determined the intensity and direction of the effect of pre-existing immunity in the population on the pathogen's ability to persist beyond the initial epidemic following its introduction.

## 1. Introduction

The infusion of modern ecological theory into epidemiology was initiated in the 1950s [1–3]. Subsequently, demographic factors controlling the fate of pathogens in animal populations have been identified in the context of zoonotic reservoirs [4–6] and wildlife conservation [1–3]. Two distinct mechanisms of pathogen extinction have been proposed theoretically and explored in various natural systems [4–6]. First, invasion thresholds are directly derived from the basic reproduction ratio (*R*_0_): deterministic models predict that a minimum density or proportion (depending on the mode of transmission) of susceptible individuals is required for a given infection to spread. However, even above this threshold epidemics may still fail to occur due to stochastic processes. A classical prediction from branching process theory is that, given *R*_0_ > 1, the probability that a single infectious individual in a naive population gives rise to an epidemic is equal to 1 – 1/*R*_0_ [7].

Second, stochastic models also predict that even when a pathogen successfully spreads in a population, it may not persist indefinitely. A relationship between population size and probability of extinction for endemic diseases was first proposed by Bartlett [8]. Combining case reports of measles in non-*naive* human populations and stochastic models with a metapopulation structure, Bartlett proposed that measles virus was more likely to ‘fade out’ in communities below a ‘critical community size’ (CCS). Many authors have subsequently confirmed that measles and other viruses resulting in acute infections in humans are more likely to fade out in smaller communities, but persist at the metapopulation level through migration [9]. Over time, CCS has become a pervasive concept through human and wildlife epidemiology. The abbreviation CCS is now often used as a general term for any population threshold for disease persistence [4] and its definition has been broadened to apply variously to population density or size (e.g. [6,10–12]). However, unlike invasion thresholds, which are simple functions of *R*_0_, the CCS is an ill-defined quantity. As underlined by Conlan *et al*. [9], CCS estimation is sensitive to the chosen measure of persistence as well as the detailed assumptions of the stochastic model used for inference; these caveats make it difficult to compare CCS estimates between studies.

The CCS was originally defined in human populations with near-continuous birth. Substantial seasonal variation in human birth rates (for example, in sub-Saharan Africa) was recently shown to have significant effects on the periodicity, magnitude and timing of measles epidemics [13]; however, its effect on CCS has not been explored. Seasonality of life-history traits and behaviour are important drivers of wildlife infectious disease dynamics [14], yet also have not been considered when estimating CCS in wildlife populations. The timing of birth in wildlife is usually tightly controlled by seasonal cycles in resource availability or climate [14]. Modelling studies using deterministic frameworks have explored the effect of seasonal reproduction of wildlife hosts on infection cycles for a variety of pathogens, including macroparasites [15], possum tuberculosis [16], house finch conjunctivitis [17], rabbit haemorrhagic disease [18,19], vole cowpox [18,20] and raccoon rabies [21,22]. Only one of these models considered disease extinction [18], but the effect of stochastic fade-out on persistence and CCS was not explored. Given the importance of birth pulses in shaping infection cycles in wildlife populations, we hypothesize that these cycles could result in pathogen extinction, thus affecting the CCS. Herein, we use a simple stochastic epidemiological model to investigate the effects of an annual seasonal birth pulse on pathogen persistence and extinction.

Specifically, we ask how host life-history traits (lifespan and shape of the seasonal birth pulse) and infection parameters (infectious period and basic reproduction ratio) interact to determine the persistence of a pathogen following its introduction in a closed population. We review published and unpublished birth pulse data across various species to motivate the structure of our demographic model. We then present results of stochastic simulation series over a range of parameter values and discuss our findings in the context of the concept of CCS.

## 2. Material and methods

### (a) Birth pulse function and empirical validation

Most species display seasonal variations in mating and births, often marked by one or two yearly peaks. In humans, births occur throughout the year and variations can be approximated well by sine functions [13,23]. By contrast, many wild mammalian species give birth only during a limited period of time each year, which has led to the common use of a step function (equal to zero for several months) to describe seasonal birth rates in mathematical models [15,20,21]. At its most extreme, all yearly births are assumed to occur simultaneously in an instantaneous pulse [16,24]. Continuous (double-logit) step functions have also been used to reduce the dynamic artefacts caused by discontinuous step changes [17]. However, even in species with a short breeding season, there is temporal variation in birth rates, so we would expect step and sine functions to be the two ends of a spectrum. We investigated empirical support for an alternative mathematical description of birth pulses that would fill the gap between those two extremes. Mathematically, a pulse is commonly modelled as a Dirac delta function that is the limit of the Gaussian function 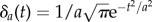 when *a* → 0. We modified the latter to make it periodic using a cosine function, leading to the following *per capita* birth rate:2.1
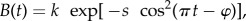
which has period of 1 time unit (here, 1 year); we refer to this as the periodic Gaussian function. This function has three parameters, which all have relevant biological interpretations: *k* is a scaling factor proportional to the annual *per capita* birth rate, *φ* controls the phase (i.e. the timing of the peak of the birth pulse) and *s* controls the bandwidth (i.e. the duration of the birth pulse). Greater values of *s* result in higher and narrower peaks, which can be interpreted as more synchronous births in the population. In the absence of a birth pulse (*s* = 0), we set the birth rate to a constant. In the following, we refer to *s* as the synchrony parameter. See the electronic supplementary material, appendix 2.1 for more detail about the function.

To compare this function with the sine and step functions, we searched the literature for published data on the timing of births in wild mammals. We collected reports of observed numbers of births by day, week or month, covering the whole period of reproduction for the populations of species considered (electronic supplementary material, appendix 1). We excluded species with two or more birth peaks within a year, as well as datasets that were either too small (fewer than 10 births recorded) or aggregated from diverse locations, resulting in a blurred seasonal signal.

We fitted a total of three birth rate functions to each dataset by maximum likelihood. The functions chosen were periodic Gaussian function, cosine function and step function. For a given model and a given dataset, the likelihood was calculated as the multinomial probability of the distribution of observed births, given the expected proportions of births obtained by integrating the birth rate over that time step. For each dataset, the three fitted models were ranked according to Akaike's information criterion (AIC); we used the second-order variant of AIC which accounts for finite sample size [25]. See the electronic supplementary material, appendix 2.2 for more information. All calculations were performed with the R software version 3.0 [26]. For each dataset *i* and each model *j*, we then calculated the AIC difference 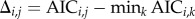, which indicates the relative support for each model [25].

### (b) Dynamic model

We model a hypothetical population with an annual birth pulse as given in equation (2.1), and assume a constant death rate *m.* In order to maintain a stationary population size from year to year, we re-wrote the scaling coefficient *k* as a function of *m* and *s*, so that the integral of *B*(*t*) over a period of 1 year is equal to *m* (see the electronic supplementary material, appendix 2.1). As a result of this assumption, the value of the death rate *m* also determines the birth rate; to reflect this, we will refer to *m* as the ‘turnover rate’. Additionally, 1/*m* represents the average lifespan in the population. Given the values of *m* and *s*, the phase of the birth pulse *φ* and the yearly average of the population size *ν*, we can calculate the population size at the point in the cycle corresponding to *t* = 0 to initiate simulations (electronic supplementary material, appendix 2.1).

At the start of the annual cycle (*t* = 0), we introduce an individual infected with a directly transmitted pathogen. The spread of infection is assumed to follow a classical SIR (susceptible–infectious–recovered) model: there is no incubation period, susceptible individuals get infected by direct contact with infectious individuals at rate *β I/N*, recovery occurs at a constant rate *γ* and recovery results in lifelong immunity. In addition, we assume that the infection is not lethal, which means that the overall population dynamics are the same with or without the pathogen.

First, we present a deterministic version of the model, defined by the following set of differential equations:
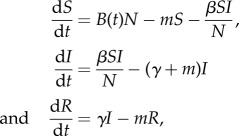
where *S*, *I* and *R* are the densities of susceptible, infected and recovered individuals respectively, *N* = *S* + *I* + *R* is the total population density, and *B*(*t*) is the seasonal *per capita* birth rate from equation (2.1). Table 1 lists the model parameters and the ranges of values explored in this study. The pathogen's basic reproduction ratio is *R*_0_ = *β*/(*m* + *γ*). Note that transmission is modelled as a frequency-dependent function (i.e. proportional to the prevalence of infection *I*/*N*), so *R*_0_ is independent of the population size. We considered two variants on this model. First, to tease apart the potential importance of increases in overall population size versus increases in the susceptible pool, we considered a model with a seasonal death rate matching the birth rate that allowed only the susceptible portion of the population to increase; it produced results qualitatively similar to those obtained with the main model (see the electronic supplementary material, appendix 6). Second, in order to tease apart frequency versus density dependence, we considered a density-dependent transmission function *β'SI* with constant death rate as in the original model (see the electronic supplementary material, appendix 7).

**Table 1.** List of symbols used in the model.

symbol	description	values explored
*S*(*t*)	susceptible individuals	—^a^
*I*(*t*)	infected individuals in population	*I*(0) = 1
*R*(*t*)	recovered individuals	—^a^
*B*(*t*)	birth rate *per capita*	—^b^
*ν*	yearly average population size	10^2^–10^5^
*p*	proportion of immune individuals in population at *t* = 0	0–0.75
*m*	death rate or turnover rate	0.1–3 yr^−1^
*s*	birth pulse synchrony	0–100
*φ*	phase of the birth pulse	−*π*/2–*π*/3
*k*	scale of the birth pulse	—^c^
*γ*	recovery rate	1–52 yr^−1^
*R*_0_	basic reproduction ratio	4
*β*	transmission rate	—^d^

^a^*S*(0) and *R*(0) are functions of *ν*, *p* and *φ* (see text).

^b^*B*(*t*) = *k* exp[−*S* cos^2^{*π t* − *φ*)].

^c^*k* is a function of *m* and *s*.

^d^*β* = *R*_0_ (*m* + *γ*).

The deterministic model was solved numerically using the deSolve package [27] in R. Given the values for *m*, the initial population size *N*(0) was calculated to ensure a yearly population average equal to the chosen value *ν* (see the electronic supplementary material, appendix 2.1). Infection was seeded with *I*(0) = 1, and the rest of the population was split between naive, *S*(0) = (1 − *p*) [*N*(0) − 1], and immune, *R*(0) = *p* [*N*(0) − 1], with 0 ≤ *p* < 1 representing the fraction of the population immune prior to pathogen introduction (due to acquired immunity from a previous outbreak or vaccination).

The core of our study is based on an event-based, stochastic version of the model, where the three state variables (*S*, *I* and *R*) can take only integer values. Six types of events (births, deaths in each state variable, infection or recovery) occur in continuous time with probabilities proportional to their respective rates in the deterministic model. However, because of the time-dependent birth rate, we decided not to use the exact Gillespie algorithm [28] as it can generate long time steps when event rates are low. Instead, we implemented an adaptive time-step algorithm [29], with a maximum step size of less than 1 day (see the electronic supplementary material, appendix 3 for a complete description). During a time step *δt*, the number of events of each type *i* = {1, … ,6} is drawn from a Poisson distribution with mean *r_i_δ_t_*, where *r_i_* is the rate of event type *i*, for example *βSI*/*N* for an infection. If more events occur than are feasible (e.g. more recoveries than there are currently infected individuals), the time step is halved and the new Poisson-distributed random numbers are drawn.

The stochastic model was implemented in R, using the same range of parameter values and initial conditions as for the deterministic model and explored a full factorial set of model parameters. For each set of parameter values and initial conditions, we ran 1000 simulations for a duration of 10 years. This arbitrary time limit was chosen to allow several seasonal cycles to occur, while keeping in mind that our assumptions of constant parameters and closed populations would not be relevant in nature over long periods of time. We estimated the probability of pathogen extinction as the proportion of simulations that reached the state *I* = 0, and we recorded the time of extinction. Combined with average infectious periods of at most 1 year, this gives us a reasonable measure of pathogen persistence following a single introduction event. Failure of an epidemic to ‘take off’ (result in sustained transmission), which is expected to occur with a probability equal to 1/*R*_0_ [7], was recorded separately to post-epidemic extinctions, using a threshold of five transmission events before extinction. This value gave results consistent with the theoretical expectation across the wide range of parameter values. In addition to the figures in the main text, the electronic supplementary material, appendices 5, 6, 7 and 9 contain more complete graphs showing interactions between parameters, as well as a global sensitivity analysis, which confirms statistically the complexity of these interactions.

## 3. Results

### (a) Empirical validation of birth pulse function

**Figure 1.**
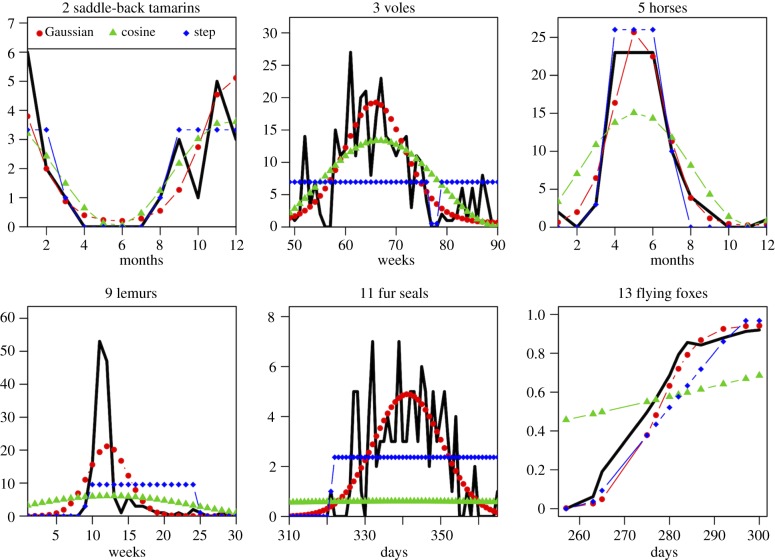
Observed and predicted births for six datasets. Black solid lines show data (numbers of births, except dataset 13: proportion of females with pups); coloured symbols show the three fitted models. Numbers preceding common names refer to datasets listed in the electronic supplementary material, table S1.

For each of the 18 datasets, we rank our three models using AIC; the results are shown in the electronic supplementary material, appendix 1. The periodic Gaussian function ranked highest with 17 datasets. For the remaining dataset (2), the cosine function is ranked first but the periodic Gaussian function still receives substantial support (ΔAIC = 2.7). The step function (which is the most commonly used one in modelling studies) receives very little support across all but one dataset. Plots of the observed and predicted dynamics show that the periodic Gaussian birth rate generally reproduces the shape of the birth distribution quite well (figure 1; electronic supplementary material, figure S2). Discrepancies occur with datasets that display a small number of births on either side of the main peak: the fitted model produces a wider and lower peak as a result (e.g. dataset 9 in figure 1).

Across our 18 datasets, the maximum-likelihood estimates of the synchrony parameter *s* range from 2.4 to 227, with a median of 30. To put these values into biological context, electronic supplementary material, figure S3 shows the duration of the birth pulse, defined arbitrarily as the period when 95% of yearly births are predicted to take place, as a function of synchrony *s*.

### (b) Demographic dynamics with periodic Gaussian birth rate

Using the periodic Gaussian birth rate from equation (2.1) scaled with the turnover rate *m* to ensure a stationary population size from year to year, the deterministic population dynamics are given by3.1
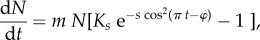
where *K_s_* is a normalization factor that depends on *s* only (see the electronic supplementary material, appendix 2.1). Numerical solutions of equation (3.1) show that *N*(*t*) follows asymmetrical annual cycles with the peak *N*(*t*) occurring after the birth pulse peak. A ‘tighter’ birth pulse (increased synchrony of births occurring over shorter duration, represented by higher values of *s*) generates greater amplitude of population cycles with a shorter time lag (electronic supplementary material, figure S4). Increasing the turnover (*m*) while keeping the average population size constant also results in oscillations of greater amplitude (electronic supplementary material, figure S5).

Stochastic simulations of this simple time-forced birth–death process enable us to assess the probability of a population crash across the {*m*, *s*, *ν*} parameter space. At the higher end of the relevant parameter region (where higher amplitude oscillations are expected), with a rapid turnover *m* = 2 yr^–1^ (equivalent to an average lifespan of six months) and a tight birth pulse *s* = 100 (representing 95% of births occurring within 33 days), average population sizes as low as *ν* = 100 crash with a frequency of only 1% within 10 years (electronic supplementary material, figure S6).

### (c) Infection dynamics and critical community size

Using our stochastic SIR model, we assess the ability of the pathogen to invade and persist following the introduction of a single case into a closed population. We distinguish between three categories of extinction occurrences: failure to take off (fewer than five cases in total), epidemic burnout following the first wave of infection (with a cut-off time of two years post introduction) and endemic fade-out (after the pathogen has persisted for at least two seasons). We focus on the latter two, having confirmed that failure to take off occurs with a probability of 1/*R*_0_, as predicted by branching process theory (see §2*b*).

**Figure 2.**
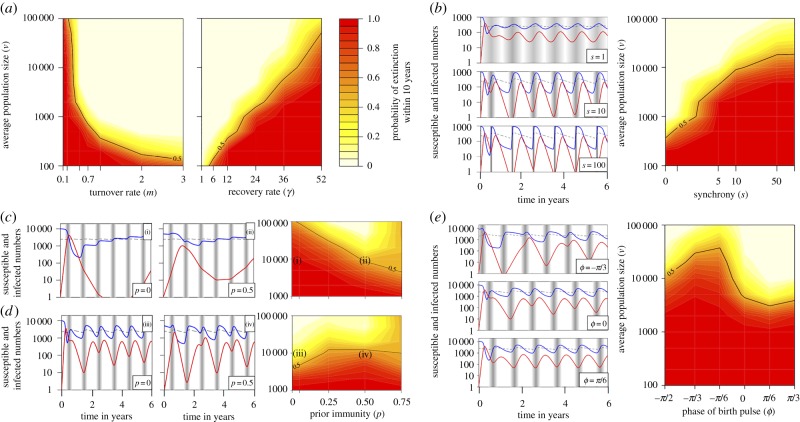
Effect of various parameters on the dynamics and persistence of infection. Contour plots show probability of pathogen extinctions within 10 years of introduction (conditional on successful invasion) as a function of average population size (*ν*) according to the scale in (*a*). The black line shows the CCS, defined as the population size resulting in 50% of pathogen extinction within 10 years. For each combination of parameter values, 1000 stochastic simulations were run. Line plots show deterministic dynamics, with the numbers of susceptible *S*(*t*) in blue and infected *I*(*t*) in red; the dashed line shows the threshold value *N*(*t*)/*R*_0_ for the number of susceptible individuals over which the infection spreads (d*I*/d*t* > 0); the width of the shaded vertical bars reflects the duration and intensity of seasonal births, *B*(*t*). (*a*) Effect of the turnover rate (*m*) and recovery rate (*γ*) in a population with a constant birth rate (*s* = 0). Parameter values: *γ* = 12 yr^−1^ (left), *m* = 1 yr^−1^ (right), *R*_0_ = 4, *φ* = 0. (*b*) Effect of synchrony parameter *s*. Parameter values: *m* = 1 yr^−1^, *γ* = 12 yr^−1^, *R*_0_ = 4, *φ* = 0. (*c*,*d*) Effect of prior immunity (*p*), comparing no prior immunity (left) with 50% of the population initially immune (right). Inset labels (i–iv) show the combinations of parameter values used in the corresponding deterministic plots. Parameter values: (*c*) *s* = 10, *m* = 0.1 yr^−1^, *γ* = 6 yr^−1^, *R*_0_ = 4; (*d*) *s* = 10, *m* = 0.5 yr^−1^, *γ* = 12 yr^−1^, *R*_0_ = 4. An increase in *p* can shift the epidemic peak closer to the next birth pulse and rescue the pathogen (*c*) or on the contrary, shift the epidemic peak from before the birth pulse to after it, resulting in deeper post-epidemic trough (*d*). (*e*) Effect of the phase of the birth pulse (*φ*). The three values of *φ* (−*π*/3, 0 and *π*/6) shown correspond to lags of two, six and 10 months from time of pathogen introduction until the next birth pulse peak. Parameter values: *s* = 10, *m* = 0.5 yr^−1^, *γ* = 12 yr^−1^, *R*_0_ = 4.

Conditional on successful invasion and in the presence of a constant birth rate throughout the year (*s* = 0), the probability of pathogen extinction is strongly influenced by demographic turnover *m* and recovery rate *γ*. In general, for a given population size *ν* and a given basic reproduction ratio *R*_0_, the probability of extinction increases with the rate of recovery *γ* and decreases with the turnover rate *m* (figure 2*a*). The population size itself has a clear positive effect on persistence. For the sake of clarity, we follow Bartlett [8] and define the CCS as the average annual population size with even odds of pathogen persistence after 10 years. A shorter time or a greater probability of extinction would result in lower CCS estimates, but the qualitative trends would remain the same: basically, the CCS decreases when the turnover *m* is higher or the recovery rate *γ* is lower (figure 2*a*). We then use these simple patterns as a background to study the effect of birth pulse synchrony on pathogen persistence and CCS.

All other parameters being fixed, increasing the birth pulse synchrony (*s*) concentrates the same number of births over a shorter time period, which amplifies oscillations in the deterministic model and tends to increase the probability of pathogen extinction (figure 2*b*). With more acute infections (i.e. higher recovery rates), a tight birth pulse (say *s* = 100) can increase the CCS by a factor of 40 compared with a constant birth rate (figure 2*a*). The quantitative effect of *s* on the CCS is generally weaker in longer-lived host species (i.e. with lower turnover *m*; electronic supplementary material, figure S7).

**Figure 3.**
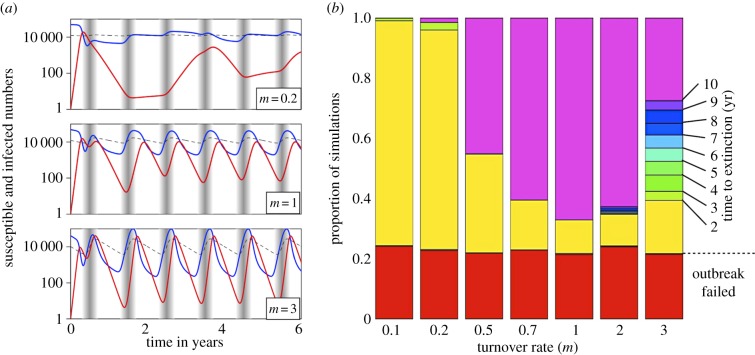
Effect of turnover rate *m* on the dynamics and persistence of infection with a birth pulse *s* = 10. (*a*) Deterministic dynamics for three values of *m* (0.2, 1 and 3 yr^−1^ from top to bottom), with the numbers of susceptible *S*(*t*) in blue and infected *I*(*t*) in red; the dashed line shows the threshold value *N*(*t*)/*R*_0_ for the number of susceptible individuals over which the infection spreads (d*I*/d*t* > 0); the width of the shaded vertical bars reflects the duration and intensity of seasonal births, *B*(*t*). (*b*) Stacked histograms of time to pathogen extinction in seven series of 1000 stochastic simulations run for 10 years, with increasing values of *m* along the horizontal axis. Red bars show the proportion of simulations with no outbreak (extinction after fewer than five infection events). Parameter values: *s* = 10, *γ* = 12 yr^−1^, *R*_0_ = 4, *ν* = 50 000.

A closer look at interactions between parameters and model dynamics reveals an unexpected pattern. In the presence of a marked birth pulse, we observe a non-monotonic effect of the turnover rate *m* on pathogen persistence (figure 3). As already mentioned, the low turnover rate associated with longer-lived species (for example, the primate, ungulate and bat datasets) favours epidemic burnout, typically within 2–3 years of introduction. However, conditional on survival past the first post-epidemic trough, endemic persistence is very likely, as the system settles down to low-amplitude oscillations (as predicted by the deterministic model; figure 3). Increasing the turnover rate has two opposite effects. On the one hand, by providing more naive offspring in the first post-epidemic birth pulse, it reduces the probability of a rapid burnout. We call this a ‘rescue effect’, a term borrowed from metapopulation biology [30]. On the other hand, by generating cycles of greater amplitude, it creates deeper annual troughs (visible in the deterministic model in figure 3), which in turn increases the probability of stochastic fade-out. Hence, everything else being equal, persistence is maximum in species with intermediate lifespans (for example, *m* = 1, representing an average lifespan of 1 year).

An additional factor that can modulate the effect of the birth pulse on the post-epidemic burnout is the timing of pathogen introduction in the seasonal cycle (controlled by the phase parameter *φ* in equation (2.1)). By default we assumed that the introduction of infection took place when births were at their lowest (six months before the maximum of the birth pulse, *φ* = 0). However, as shown in figure 2*e* with an average lifespan of 2 years and an infectious period of one month, a shorter lag between pathogen introduction and the next peak of the birth pulse (e.g. two months, *φ* = –π/3) can result in a dramatic increase in the CCS. The optimal phase difference for pathogen persistence (around eight months in figure 2*e*, *φ* = π/6) varies with combinations of *m* and *γ* (electronic supplementary material, figure S8). Epidemic burnout is most likely (i.e. the deepest post-epidemic trough) when the initial epidemic peak coincides with the birth pulse peak, and therefore is followed by a waning of susceptible individuals entering the population and maximal time until the ‘rescue effect’ of the next birth pulse. However, conditional to persistence beyond this first post-epidemic trough, the relative timing of the birth pulse and introduction of infection had little effect on long-term persistence.

We also considered pre-existing immunity in the population (parameter *p*) to simulate the effect of reintroduction of a pathogen after a previous outbreak and extinction, or after an immunization programme. Prior immunity reduces the probability of an outbreak, but has a highly variable effect on the CCS (which we estimated conditionally on outbreak occurrence). By reducing the effective reproduction ratio of the pathogen, prior immunity slows down the initial invasion. As a result, the birth pulse occurs earlier in the epidemic cycle, which can either shift the epidemic peak closer to the next birth pulse and rescue the pathogen (figure 2*c*) or, on the contrary, shift the epidemic peak from before the birth pulse to after it, resulting in deeper post-epidemic trough (figure 2*d*). In line with previous points, other parameters that affect the relative timing of the epidemic peak to the peak of the birth pulse (especially *m*, *γ* and *φ*) will affect the intensity and direction of the effect of *p* on CCS (figure 2*c*,*d*; electronic supplementary material, figure S9).

Taken together, these results suggest that the relative importance of a parameter for pathogen persistence and CCS is dependent on the respective variance of other parameters, which is largely arbitrary in this study. Hence, a sensitivity analysis will be most informative in the context of specific systems where more information on parameter values is available. We have provided an extensive series of plots in the electronic supplementary material that show more details of parameter interactions and nonlinear patterns.

### (d) Density-dependent model

A model with density-dependent transmission gave results similar to the frequency-dependent model over the majority of the parameter space, indicating little overall effect of density-dependent transmission on persistence (electronic supplementary material, figure S11). However, at extreme values of *m* and *s,* greater fluctuations in total population size resulted in amplified peaks and troughs of transmission, increasing the likelihood of endemic fade-out (i.e. a higher CCS; electronic supplementary material, figure S12).

## 4. Discussion

Growing concern about emerging zoonotic infections has stimulated research effort to model the dynamics of pathogen spillover from wildlife into human populations [31–33]. However, these approaches mostly ignore the dynamics of infection in the reservoir host. This motivated our study into factors that drive the persistence and extinction of pathogens in wildlife host populations, focusing on seasonal birth pulses, a feature common to many animal species.

We identified data on seasonal birth rates for a range of mammalian species. Whereas numerous studies report the period over which births take place, few provide the time series of birth numbers required to calculate birth rates for such study, even though appropriate data probably exist in raw form. We assessed alternative mathematical functions for the birth pulse using 18 datasets. Our results suggested that the binary step function used in most published models was not a good representation of real birth pulses and the ‘periodic Gaussian’ function may be preferable for this purpose. Estimates of the key parameter controlling the tightness of the birth pulse (*s*, for synchrony) across available datasets of birth pulses have not previously been quantified, yet span two orders of magnitude and have significant effects on the infection dynamics.

Our stochastic SIR model with annual birth pulses showed that tighter birth pulses tend to drive pathogen extinction by creating large amplitude oscillations in prevalence. In addition, tighter birth pulses result in the population size being lower for a greater proportion of the year, leading to an increased likelihood of stochastic fade-out. The effect of *s* was stronger in species with higher demographic turnover, and for pathogens with shorter infectious periods and density-dependent transmission. Interestingly, in the presence of a birth pulse, invasive pathogens are predicted to be most likely to persist in host species with intermediate turnover (measured as the average number of births and deaths per year): long-lived species with small birth pulses tend to experience a single epidemic, which dies out; by contrast, short-lived species with a higher birth pulse can maintain the pathogen for a few seasons but with a pattern of annual peaks and troughs, which often results in stochastic extinction of the pathogen. Early, post-epidemic fade-outs are also affected by the timing of pathogen introduction relative to the birth pulse, as well as pre-existing immunity in the population (e.g. from a recently extinct outbreak).

Empirical estimation of CCS in wildlife remains limited [4]. A few studies have used mathematical models to analyse the role of wildlife reservoir dynamics in the occurrence of zoonotic spillover events in humans, highlighting factors affecting pathogen persistence in reservoir host populations. In particular, plague outbreaks have been linked to the metapopulation structure of the rat reservoir in Europe [34] and gerbils in Kazakhstan [35,36]. However, as underlined by Heier *et al*. [36], estimating the effect of host abundance on the persistence of infection is more complicated than determining thresholds for pathogen invasion. George *et al*. [24] showed that seasonal patterns of hibernation and highly synchronous reproduction in American big brown bats (*Eptesicus fuscus*) played a crucial role in the persistence of rabies virus in that host, but their study only considered large populations, hence offering little insight into CCS. Other ecological factors, such as contacts between multiple host species, have been proposed to contribute to pathogen persistence in wildlife by increasing the effective community size [6,37].

Apart from contributing to theoretical understanding of viral dynamics and persistence, the notion of the CCS has practical applications in wildlife population management. For example, vaccination is often difficult or impractical in wildlife, and it is often recommended in combination with reduction of population size by culling susceptible animals (discussed in [38–41]). However, our model suggests that prior herd immunity can increase the CCS in some circumstances. Sufficient life-history data were not available for all of the seasonal birth pulse datasets presented here to estimate the CCS required for pathogen persistence over a range of infectious periods. However, we provide two specific examples to demonstrate the real-life utility of this model when data are available. First, our results indicated that the effect of birth pulse synchrony on the CCS was more pronounced in shorter-lived host species (i.e. with higher turnover *m*), such as Townsend's vole (*Microtus townsendii*, dataset 3, *m* ≈ 3.3 [42]). Even with a relatively low degree of synchrony (*s* = 3.3, representing 95% of births occurring within 7.8 months), the presence of the birth pulse in *M. townsendii* increases the CCS for a pathogen with an infectious period of one month (*γ* = 12) from less than 200 to almost 10 000 individuals (electronic supplementary material, figure S13). An even greater increase in CCS is expected for pathogens with more acute infectious periods (electronic supplementary material, figure S7). Second, we consider the grey-headed flying fox (*P. policocephalus*, dataset 13), which has a highly synchronous seasonal birth pulse (*s* = 130, representing 95% of births within 28 days), but a low turnover rate (*m* = 0.14 [43]), which moderates the effect of the birth pulse (figure 2*b*). Our model predicts that pathogens with an infectious period of less than approximately six weeks (*γ* ≥ 8) could not persist in a naive population with this turnover rate and degree of synchrony (electronic supplementary material, figure S14*a*). This raises questions regarding the dynamics of pathogens with short infectious periods, such as Hendra virus (*γ* ≈ 52) [44] within populations of this species. The inclusion of pre-existing immunity at 50% (equivalent to Hendra virus seroprevalence rates commonly observed in this species [44]) resulted in greater persistence, though still only to *γ* ≈ 20 (electronic supplementary material, figure S14*b*). This suggests that other factors important in viral persistence in this system are absent from our model; for example, age-structure, metapopulations, multi-host systems, within-host persistence and waning immunity.

Our model provides theoretical insights into the effect of seasonal birth pulses on pathogen dynamics in wildlife populations and a basis for further extension. For example, one extension would be to consider a metapopulation framework, which would allow recurrent introduction of the pathogen. Interestingly, our model predicts that prior immunity can favour the persistence of some pathogens by dampening the secondary outbreak dynamics, a phenomenon related to the ‘epidemic enhancement’ proposed by Pulliam *et al*. [45] and applied within a metapopulation framework by Plowright *et al*. [44]. There is ongoing debate on the effects of habitat fragmentation on the persistence of infectious diseases in wildlife [46]. A landmark study by Swinton *et al*. [12] concluded that the fragmented metapopulation structure of harbour seals around the North Sea was responsible for the rapid fade-out of a deadly outbreak of phocine distemper virus in 1988. Recent examples indicate that existing habitat fragmentation and variations in population sizes could be used to hinder the threat from infectious diseases to endangered wildlife, including rabies in the Ethiopian wolf [47] and chytridiomycosis in amphibians [48]. Our model could also be modified to account for disease-induced death in order to investigate the role of seasonal birth pulse on the relative risks of host and pathogen extinction.

Despite growing interest in the environmental and demographic drivers of pathogen cycles in wildlife [14], the effect of these cycles on pathogen persistence has been overlooked. By incorporating an empirically motivated birth pulse function into a generic infection model, we have provided a framework to study pathogen persistence in wildlife species exhibiting seasonal births. The CCS is sensitive to demographic and pathogen-related parameters, and should be considered within an ecological context. Therefore, estimation of CCS values and their subsequent use in wildlife management practices must be treated with caution as it is likely to be highly system-dependent.

## Data accessibility

All unpublished data used in this study are included in the appendices in the electronic supplementary material.
